# The complete mitochondrial genome of the snowy-browed flycatcher *Ficedula hyperythra* (Passeriformes, Muscicapidae)

**DOI:** 10.1080/23802359.2021.1970639

**Published:** 2021-09-13

**Authors:** Nan Yang, Wei Liu, Kong Yang

**Affiliations:** a Institute of Qinghai-Tibetan Plateau, Southwest Minzu University, Chengdu, China; b Collaborative Innovation Center for Ecological Animal Husbandry of Qinghai- Tibetan plateau, Southwest Minzu University, Chengdu, China; c College of Animal & Veterinary Sciences, Southwest Minzu University, Chengdu, China

**Keywords:** Ficedula hyperythra, mitochondrial genome, next-generation sequencing

## Abstract

In this study, we sequenced and annotated the complete mitochondrial genome of *Ficedula hyperythra* (Blyth, 1843). The mitogenome of *F. hyperythra* is 16,819 bp in length and contains 13 protein-coding, 22 transfer RNA, and 2 ribosomal RNA genes. Phylogenetic analysis based on 13 concatenated protein-coding genes showed that *F. hyperythra* clustered with other Ficedula species and had a close relationship with *F. albicilla*.

The genus *Ficedula* has become a model system for studies of avian speciation, genomics, biogeography, and the evolution of migratory behavior (Moyle et al. 2015). The snowy-browed flycatcher, *Ficedula hyperythra* (Blyth, 1843) is one of species in *Ficedula* and was reported to be not monophyletic (Moyle et al. 2015). The whole mitochondrial genome (mitogenome) will be useful to better understand the phylogenetic context, genetic diversity, and geographic distribution of this species. In this study, we determined a complete mitogenome of *F. hyperythra* using next-generation sequencing technology.

The sample was collected from Leshan, Sichuan, China (28°42′N, 103°03′E). A specimen was deposited at Southwest Minzu University (Nan Yang, yangnan0204@126.com) under the voucher number SWMZU-JWY-B24101. A small amount of muscle tissue was shipped to Sangon (Shanghai, China) for genomic extraction and sequencing library construction. Whole genome sequencing was performed to generate 150 bp paired-end reads on the HiSeq 2500 platform (Illumina). After quality control, *de novo* assembly of clean reads was conducted using SPAdes v3.14.1 (Prjibelski et al. 2020). Genomic features were annotated with the MITOS web server (Bernt et al. 2013). The final assembled mitogenome was deposited in GenBank of NCBI with accession number MW795347.

The mitogenome of *F. hyperythra* was 16,819 bp in length and contained 13 protein-coding genes (PCGs), 22 transfer RNA genes, two ribosomal RNA genes and one putative control region. The nucleotide composition is significantly biased 29.27% A, 33.19% C, 14.98% G, 22.55% T, with a G + C content of 48.18%. The gene arrangement and composition were identical with the mitogenome of *F. albicilla* (Zhang and Lu 2019), exhibiting a typical vertebrate mitochondrial gene arrangement. The start codon for all PCGs was ATG, except COX1, which had ATC as the start codon. We identified three different complete termination codons: AGG for COX1, TAG for ND1 and ND6, TAA for ND2, COX2, ATP8, ATP6, ND3, ND4L, ND5, and Cytb. For COX3 and ND4, the termination codon was incomplete (T–).

Phylogenetic analysis for *F. hyperythra* and the other 24 Muscicapidae species was conducted by concatenating nucleotide sequences of 13 PCGs. One species of Mohoidae (*Moho braccatus*) was used as an outgroup. Multiple sequence alignment was performed using MAFFT v7.475 (Katoh and Standley 2013). A maximum-likelihood phylogenetic tree was constructed using IQtree v1.6.12 (Nguyen et al. 2015) with 1000 bootstrap replicates and TVM + F+R4 substitution model suggested by modelFinder (Kalyaanamoorthy et al. 2017). The ML phylogenetic tree illustrated that our sample clustered with other Ficedula species and had a close relationship with *F. albicilla* (Figure 1).

**Figure 1.**
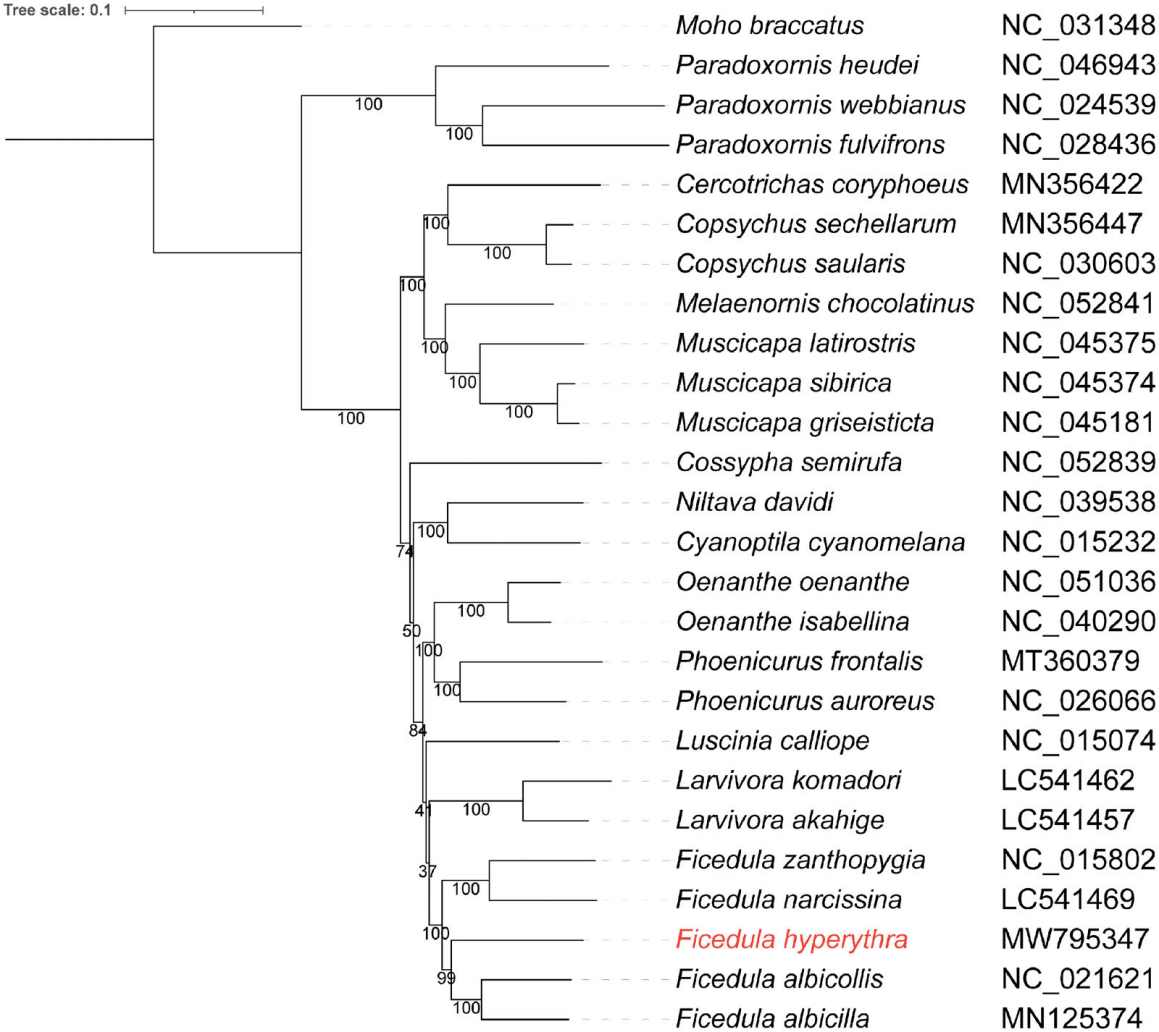
Phylogenetic tree of *F. hyperythra* and additional 24 Muscicapidae species with one outgroup constructed using the maximum likelihood (ML) method based on 13 protein-coding genes.

## Data Availability

The genome sequence data that support the findings of this study are openly available in GenBank of NCBI at https://www.ncbi.nlm.nih.gov/ under the accession MW795347. The associated BioProject, SRA, and Bio-Sample numbers are PRJNA753463, SRR15403527, and SAMN20695163 respectively.
